# Psychiatric inpatient bed capacity and suicide mortality in Sweden: a nationwide ecological study

**DOI:** 10.1016/j.lanepe.2026.101675

**Published:** 2026-04-08

**Authors:** Jonas Berge, Måns Gerle, Sara Lindström

**Affiliations:** a Department of Clinical Sciences, Lund, Psychiatry, Lund University, 22185, Lund, Sweden; b Department of Psychiatry, Skåne University Hospital, Malmö, Sweden; c Department of Psychiatry, Skåne University Hospital, Lund, Sweden

**Keywords:** Suicide mortality, Suicide prevention, Psychiatric inpatient care, Health service utilization, Sweden, Public health psychiatry

## Abstract

**Background:**

Suicide is a leading cause of premature mortality worldwide, but the impact of system-level mental health resources remains unclear despite the central role of psychiatric inpatient care and declining bed capacity. Prior research has yielded inconsistent findings with limited control for confounding. We aimed to estimate the effect of psychiatric inpatient bed availability on suicide mortality in a universal, publicly dominated mental health-care system.

**Methods:**

We conducted a nationwide ecological study using data from 20 Swedish counties between 2015 and 2024. Suicide mortality was obtained from the Cause of Death Register. Psychiatric inpatient bed availability and county-level healthcare budget allocation were extracted from Swedish Municipalities and Regions. Pooled cross-sectional quasi-Poisson regression models, fixed effects models, and Bayesian hybrid mixed-models were used to estimate effects between and within counties. The primary effect estimate was the percentage change in suicide mortality associated with a 10-bed increase per 100,000 inhabitants.

**Findings:**

Psychiatric bed capacity declined nationwide from approximately 30.6 beds per 100,000 inhabitants in 2015 to 24.2 in 2024. The overall between-county effect was estimated to 0.892 (95% CI: 0.827–0.961). In the primary analysis of within-county effects, each 10-bed increase per 100,000 inhabitants was associated with a 7.6% reduction in suicide mortality (rate ratio 0.924, 95% CI: 0.881–0.969; p = 0.001). In the Bayesian within-between decomposition models, only the within-county effect of psychiatric bed capacity was fully within the credible intervals (95% credible interval 0.852–0.994).

**Interpretation:**

Greater psychiatric bed availability was associated with lower suicide rates, highlighting the inverse association of inpatient care availability and suicide mortality in a high-income country. Extrapolating to Sweden's population of 10.6 million in 2024, a return to the psychiatric bed capacity of 2015 would correspond to approximately 83 fewer suicides annually, assuming that the association is causal. Safeguarding timely access to inpatient psychiatric care may be an important component of national suicide-prevention strategies.

**Funding:**

USVE grants.

**Research in context**

**Evidence before this study**

We searched PubMed using the terms *psychiatric beds*, *inpatient care*, *mental health services*, *suicide*, *suicide mortality*, and *deinstitutionalisation*. Existing evidence is highly heterogeneous. Early studies from the deinstitutionalisation era focused predominantly on patients with schizophrenia and reported mixed findings: some Scandinavian cohort studies identified increased suicide mortality associated with large reductions in inpatient capacity, whereas others found no increase or even decreases in suicide risk. More recent ecological studies examining population-level trends have produced conflicting results, including positive associations, null findings, and simulation-based thresholds (e.g., elevated suicide risk below 25 beds per 100,000 inhabitants). However, nearly all previous studies have been limited by insufficient control for confounding, reliance on aggregate cross-sectional comparisons, or being conducted in the United States—a health-care context with substantial private-sector involvement and no universal coverage, limiting generalisability.

**Added value of this study**

To our knowledge, this is the first study to estimate the association between psychiatric inpatient bed availability and suicide mortality using robust statistical methods in a universal, publicly dominated health-care system resembling most high-income countries. By exploiting within-country regional variation in budget allocation for psychiatric inpatient care and using nationwide, high-quality registers with complete population coverage, we overcome key limitations of prior observational studies. We demonstrate that an increase in inpatient capacity is associated with statistically robust and clinically meaningful reductions in suicide mortality at the population level, with consistent findings across modelling approaches.

**Implications of all the available evidence**

Taken together, the international evidence and our findings suggest that the long-term trend of reducing inpatient capacity—often justified by the expansion of community mental-health services—may carry unintended consequences for suicide risk when bed supply becomes critically constrained. While community psychiatry remains essential, maintaining adequate inpatient capacity appears fundamental for safe crisis management, timely hospitalization, and prevention of high-risk care transitions. For high-income countries undergoing continued reductions in psychiatric beds, safeguarding inpatient capacity should be considered a central component of national suicide-prevention strategies.

## Introduction

Suicide remains a major cause of premature mortality worldwide and a persistent public health challenge.^1^, ^2^, ^3^ Although global suicide mortality has declined in recent decades, progress has slowed and several regions have experienced plateaus or increases, suggesting that broader social, economic, and structural factors shape national suicide trajectories. Psychiatric inpatient care has traditionally played a central role in suicide prevention by providing intensive monitoring, crisis containment, and evidence-based treatment for individuals at acute risk. However, in most high-income countries, psychiatric services have undergone extensive deinstitutionalisation since the 1990s, resulting in sustained reductions in inpatient bed capacity,^4^^,^^5^ and continued policy shifts toward community-based and outpatient models.^6^ These reductions have raised concerns that constrained access to inpatient care may delay hospitalization, increase admission thresholds, and heighten exposure to high-risk care transitions. Despite this, suicide rates in many high-income countries have not increased proportionally, possibly reflecting compensatory effects of expanded community services and crisis interventions. While outpatient services may reduce suicide risk,^7^ the balance between inpatient capacity and community care varies across contexts, complicating the interpretation of observed associations. International evidence demonstrates substantial heterogeneity by context and analytical method. Early studies of deinstitutionalization, characterised by marked reductions in psychiatric hospital beds, often focused on patients with schizophrenia. A Swedish cohort study reported increased suicide mortality following reductions in bed capacity among patients with schizophrenia.^8^ with similar findings in Denmark, while a Finnish study found no such increase.^9^ In a later Finnish study examining a broader range of psychiatric disorders, no increase in suicide risk was observed following reductions in psychiatric beds, and suicide rates decreased for several diagnostic groups.^10^

More recent ecological studies comparing population suicide rates with psychiatric bed availability have likewise yielded inconsistent results. In the United States, Yoon and Bruckner found that reductions in public psychiatric beds were associated with increased suicide mortality at the state level between 1982 and 1998,^11^ whereas Gibbons and colleagues found no such association using data from 1999 to 2013.^12^ Finally, Lee and colleagues also failed to find such an association in Hong Kong data from 1999 to 2015. Furthermore, using a simulation-based approach, Atkinson and colleagues predicted that suicide mortality might increase when psychiatric bed availability decreases below 25 beds per 100,000 inhabitants.^13^

These mixed findings have important implications for policy, particularly given continued reductions in inpatient capacity in many high-income countries. A key limitation of the existing literature is that psychiatric bed allocation and suicide rates are closely intertwined with socioeconomic context, service organisation, and demographic factors, complicating causal inference. Insufficient control for confounding and methodological heterogeneity further limit interpretability, leaving substantial uncertainty regarding the true relationship between inpatient bed availability and suicide outcomes.

To address these issues, we applied robust statistical methods that leverage regional and temporal variation in psychiatric bed capacity and suicide rates, as well as total budget spending on psychiatric care per capita and the percentage of that budget spending allocated to outpatient services. The present study provides the first robust estimate of the effect of psychiatric inpatient capacity on suicide mortality using adequate statistical methods in a universal, publicly dominated health-care system, a context representative of most high-income countries. This study aims to estimate the association between psychiatric inpatient bed availability and suicide rates in Sweden during 2015–2024.

## Methods

### Study design

This longitudinal ecological study examined the relationship between psychiatric service capacity and suicide rates across Swedish counties from 2015 to 2024.

### Sample size

The study included all Swedish counties with available data during the study period; no formal sample size calculation was performed, as the study used nationwide administrative data.

We employed a multi-level analytical strategy combining pooled cross-sectional analyses, fixed-effects regression, and Bayesian mixed-effects modeling to examine both between-county and within-county associations. The study initially included all 21 Swedish counties but Gotland County was excluded due to missing budget data. Gotland's mean annual population (59,676) was substantially smaller than any included county (Table 1), though its suicide rate (17.3 per 100,000) and psychiatric inpatient bed capacity (25.0 per 100,000) were within the range observed across included counties, suggesting that its exclusion is unlikely to have materially biased the results. Additionally, data from 2023 to 2024 for Södermanland county were excluded due to implausible psychiatric budget figures, which if taken at face value would indicate a decrease in psychiatric inpatient budget of approximately 53% from 2022 to 2023, and 99% from 2022 to 2024 while the psychiatric inpatient beds decreased by 21% during that time period. These figures most plausibly reflect administrative error rather than true changes in budget allocation. Under this assumption, the excluded observations can be considered missing completely at random, making complete case analysis appropriate without the need for imputation. All other data were complete, resulting in 198 county-year observations for the final analytical dataset.

**Table 1:** Descriptive characteristics of included Swedish counties, 2015–2024.

County	Mean annual population	Suicide rate	Psychiatric inpatient beds	Outpatient budget (%)	Total psychiatric budget
Blekinge	158,534	17.4	28.2	55.4	3.41
Dalarna	286,273	18.5	19.7	60.9	2.93
Gävleborg	285,842	18.8	20.9	56.2	2.82
Halland	332,737	14.2	20.4	51.1	2.61
Jämtland	130,654	19.3	27.5	56.3	3.18
Jönköping	361,962	14.2	28.6	54.5	3.03
Kalmar	244,528	16.2	28.4	57.5	2.93
Kronoberg	200,016	15.0	26.0	67.1	3.37
Norrbotten	249,782	16.7	21.5	49.2	2.98
Skåne	1,375,394	14.7	27.2	58.2	2.83
Stockholm	2,367,778	13.6	34.2	71.5	3.57
Södermanland	295,969	15.4	26.1	65.3	2.89
Uppsala	383,476	14.5	22.2	57.3	2.64
Värmland	281,486	17.7	21.3	65.2	3.13
Västerbotten	272,269	12.8	27.4	58.4	3.26
Västernorrland	244,202	14.9	29.0	57.7	3.03
Västmanland	274,972	17.2	22.5	53.5	3.12
Västra Götaland	1,721,000	15.0	28.1	55.5	2.84
Örebro	302,693	14.9	24.9	60.0	3.10
Östergötland	463,356	14.5	17.6	57.3	2.91

Values represent means over the study period 2015–2024. Suicide rate is expressed per 100,000 inhabitants per year. Psychiatric inpatient beds are expressed per 100,000 inhabitants. Outpatient budget (%) denotes the proportion of the total psychiatric budget allocated to outpatient services. Total psychiatric budget is expressed in thousands of SEK per capita, adjusted to 2024 prices using the Consumer Price Index.

Data were obtained from multiple Swedish national registers and databases, all reported at the county-year level and publicly available through the Swedish National Board of Health and Welfare (NBHW), Statistics Sweden (SCB), or Swedish Municipalities and Regions (SKR). No missing data were present in the final analytical dataset. Suicide deaths (ICD-10 codes X60-X84 and Y10–Y34) were obtained from the Cause of Death Register for 2015–2024, stratified by county and year.^14^ Deaths of undetermined intent (Y10–Y34) were included as suicides due to the high prevalence of misclassified suicides in that category.^15^, ^16^, ^17^ Adult psychiatric inpatient bed capacity per 100,000 inhabitants (excluding forensic psychiatry) was obtained directly from SKR's Economic and Activity Statistics for Regions.^18^ Adult psychiatry bed data were available from 2015 to 2024, determining our study period. Total psychiatric healthcare budget and outpatient budget allocation were obtained from SKR Economic and Activity Statistics.^18^ The total psychiatric budget was adjusted to constant 2024 prices using the Consumer Price Index and expressed per capita in thousands of SEK.^19^ The outpatient budget percentage was calculated as the proportion of the total psychiatric budget allocated to outpatient services. Population data by county and age were obtained from SCB.^20^ The calendar year was included as a continuous variable to account for secular trends in suicide rates.

The primary outcome was the annual suicide rate per 100,000 inhabitants by county. The main exposures were: (1) psychiatric inpatient beds per 100,000 inhabitants, (2) total psychiatric budget per capita (thousands of SEK, CPI-adjusted to 2024 prices), and (3) percentage of psychiatric budget allocated to outpatient services. For interpretability, all bed effects are reported per 10-unit increase in psychiatric beds per 100,000 inhabitants. These data reflect total psychiatric inpatient bed capacity and do not distinguish between voluntary and involuntary care, which are delivered within the same inpatient units in Sweden.

### Statistical analysis

We employed three complementary analytical approaches to examine different aspects of the relationship between psychiatric service capacity and suicide rates. Formal model specifications with notation for all three approaches are provided in the Technical Appendix.

1. **Pooled Cross-Sectional Analysis (Between-County Effects):** This approach assessed time-invariant differences between counties by pooling suicide outcomes and population data across all years while using county-averaged values for exposure variables. This analysis examines how baseline differences in average service capacity over the study period relate to total suicide burden across counties. Exposure variables (psychiatric inpatient beds per 100,000 inhabitants, outpatient budget percentage, and total psychiatric budget per capita) were included as county means averaged over 2015–2024. Psychiatric inpatient beds were divided by 10 to provide more interpretable estimates. Calendar year was not included as time is collapsed across the study period in this specification. Unadjusted models included each exposure variable separately; the fully adjusted model included all three simultaneously.
2. **Fixed-Effects Analysis (Within-County Effects):** This approach examined how temporal changes in service capacity within counties relate to changes in suicide rates. Fixed-effects models effectively control for all time-invariant county characteristics (observed and unobserved) by using each county as its own control, focusing exclusively on within-county variation over time.^21^ This removes potential confounding from stable county-level factors such as geographic, cultural, or demographic characteristics that might influence both service provision and suicide rates. Exposure variables (psychiatric inpatient beds per 100,000 inhabitants, outpatient budget percentage, and total psychiatric budget per capita) were included as annual observations. Psychiatric inpatient beds were divided by 10 to provide more interpretable estimates. Calendar year was included as a continuous variable to account for secular trends in suicide rates. Unadjusted models included each exposure variable separately alongside the fixed effects and year trend; the fully adjusted model included all three simultaneously.
3. **Hybrid Mixed-Effects Models (Robustness Testing with Between-Within Decomposition):** As a robustness check and to provide additional insight into the source of associations, these models explicitly separated between-county and within-county effects, while also taking random effects into account.^21^ This method allowed us to test whether the fixed-effects results were robust to different modeling assumptions and to examine whether associations operated primarily through stable differences between counties or through temporal changes within counties, essentially testing both between and within-effects simultaneously, albeit at the cost of fewer degrees of freedom and thus statistical power. For each exposure variable, we created: (a) county means averaged over all available years (between-county component), and (b) annual deviations from county means (within-county component). Psychiatric inpatient beds were divided by 10 to provide more interpretable estimates. Calendar year was included as a continuous variable. Unadjusted models included each exposure variable (decomposed into between and within components) separately alongside the year trend and county-level random intercept; the fully adjusted model included all three exposure variables simultaneously.

Pooled cross-sectional models used Poisson regression with a freely estimated dispersion parameter (quasi-Poisson) and appropriately scaled standard errors to account for overdispersion. The values of the dispersion parameter ranged from 5.4 to 7.3 across the four models. Fixed-effects models employed conditional Poisson regression using the fixest package in R, with clustered standard errors at the county level.^22^ These models showed acceptable dispersion (range: 1.27–1.29, p > 0.30 for overdispersion tests), supporting Poisson specification. Bayesian models were implemented using the brms package with default priors, running 4 chains with 2000 iterations each (1000 warmup), using Markov Chain Monte Carlo (MCMC) methods.^23^ All models used log population as an offset term to model rates rather than counts.

We tested the Bayesian models using both Poisson and negative binomial distributions. The negative binomial models yielded extremely large shape parameters (5,000, 20,000, 2.14 × 10^13^, and 4.2 × 10^23^ across the four models), indicating that substantial overdispersion was not present and that Poisson distribution assumptions were appropriate for these data. Additionally, negative binomial models experienced convergence issues while Poisson models converged successfully, further supporting our choice of Poisson specification. Model diagnostics for the Bayesian models, as well as assessment of autocorrelation and VIF for the included covariates, are reported in the Technical Appendix.

Sensitivity analyses were performed with only certain suicides (X60-X84) and deaths of undetermined intent (Y10–Y35) separated. All three analytical approaches were replicated for each of these two alternative outcome measures. We also performed sensitivity analyses with the time period limited to 2015–2019 to assess whether the results were influenced by changes associated with the Covid-19 pandemic, as well as a sensitivity analysis with Stockholm County (the largest county, accounting for about 16.8 percent of the population) excluded.

Statistical significance was assessed at α = 0.05 for frequentist models, while Bayesian models report 95% credible intervals (2.5th to 97.5th percentiles). All analyses were conducted using R version 4.4.2.^24^ This study used publicly available administrative data and did not require ethical approval.

This study is reported in accordance with the STROBE (Strengthening the Reporting of Observational Studies in Epidemiology) reporting guidelines.

### Ethics approval

The study used de-identified, routinely collected population register data. According to Swedish law and the Swedish Ethical Review Authority, secondary analyses of such data do not require additional ethical review when no individual can be identified. All data handling complied with Swedish data protection regulations. No personal identifiers were accessible to the research team at any stage.

### Role of the funding source

The funders had no role in study design, data collection, data analysis, data interpretation, writing of the report, or the decision to submit the paper for publication. None of the authors has been paid to write this article by a pharmaceutical company or other agency.

## Results

The analytical sample comprised 198 county-year observations from 20 Swedish counties over 2015–2024. The mean annual suicide rate was 15.0 per 100,000 inhabitants (SD = 0.7). Psychiatric inpatient bed capacity averaged 27.3 beds per 100,000 inhabitants (SD = 2.1), showing substantial variation across counties and years. Total psychiatric budget averaged 3.0 thousand SEK per capita (SD = 0.1), while the average outpatient budget percentage was 61.2% (SD = 1.3). Descriptive characteristics of all included counties are presented in Table 1.

**Fig. 1:**
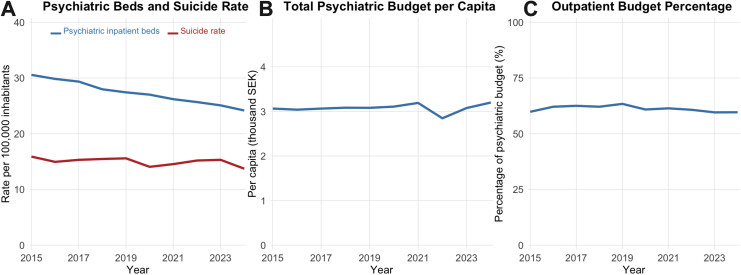
Temporal trends in psychiatric service capacity and suicide rates in Sweden, 2015–2024.

Over the study period, psychiatric bed capacity showed a consistent nationwide declining trend, decreasing from approximately 30.6 beds per 100,000 inhabitants in 2015 to 24.2 in 2024 (Fig. 1). Total psychiatric budget per capita remained relatively stable around 3.0–3.2 thousand SEK, with some fluctuation. The psychiatric outpatient budget percentage showed modest variation, ranging between 59.6 and 63.4%.

**Fig. 2:**
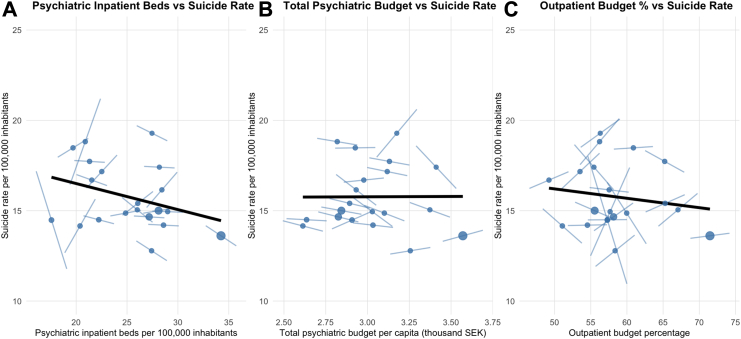
Pooled cross-sectional relationships between psychiatric service capacity and suicide rates across Swedish counties. County-level associations showing suicide rates per 100,000 inhabitants versus (A) psychiatric inpatient bed capacity per 100,000 inhabitants, (B) total psychiatric budget per capita (thousands of SEK), and (C) percentage of psychiatric budget allocated to outpatient services. Each point represents a county's average over 2015–2024 (n = 20 counties). Black lines show overall between–county relationships using pooled data; blue lines show individual county trajectories representing within-county associations. Negative slopes in panel A indicate that counties with higher bed capacity tend to have lower suicide rates, both between counties and within counties over time.

The pooled cross-sectional analysis using quasi-Poisson regression to account for overdispersion indicated an inverse association between psychiatric beds and suicide rates between counties (Table 2). Each 10-unit increase in psychiatric beds per 100,000 inhabitants was associated with a 10.8% reduction in suicide rates in the unadjusted model (RR = 0.892, 95% CI: 0.827–0.961, p = 0.008). In the adjusted model including all psychiatric service variables, the protective effect of beds remained significant (RR = 0.884, 95% CI: 0.790–0.989, p = 0.047), while outpatient budget percentage and total psychiatric budget showed no significant associations (p = 0.515 and p = 0.456, respectively). The unadjusted rate ratios are shown in Fig. 2 along with individual within-county regression lines for visualisation.

**Table 2:** Pooled cross-sectional quasi-Poisson Regression (Between-Regions Effects).

Variable	Unadjusted		Adjusted	
	RR (95% CI)	p value	RR (95% CI)	p value
Psychiatric inpatient beds	0.892 (0.827–0.961)	0.008	0.884 (0.790–0.989)	0.047
Outpatient budget percentage	0.994 (0.988–1.000)	0.071	0.996 (0.986–1.007)	0.515
Total psychiatric budget	0.897 (0.782–1.029)	0.137	1.098 (0.865–1.393)	0.456

Rate ratios (95% confidence intervals, CI) from quasi-Poisson regression models examining between-county differences in suicide rates. Models use county-averaged exposure variables (2015–2024) and pooled suicide outcomes across all years. A freely estimated dispersion parameter and appropriately scaled standard errors were included to account for over-dispersion (dispersion parameters 5.4–7.3). Effects represent how counties with different average service capacity levels differ in their overall suicide burden. Psychiatric inpatient beds per 10-unit increase per 100,000 inhabitants; outpatient budget percentage per 1% increase; total psychiatric budget per 1000 SEK increase per capita.

The conditional Poisson regression with county fixed effects demonstrated significant within-county effects for psychiatric beds (Table 3). In Model 1, each 10-unit increase in beds was associated with a 7.6% reduction in suicide rates (RR = 0.924, 95% CI: 0.881–0.969, p = 0.001). This effect remained significant in the fully adjusted Model 4 (RR = 0.920, 95% CI: 0.847–0.999, p = 0.048). Neither the outpatient budget percentage nor the total psychiatric budget showed significant within-county effects across any model specification. When using Akaike Information Criterion to compare models, Model 1 showed the best fit at 1427.4 compared to 1430.7 for Model 4, and higher scores for the remaining models, indicating that there was no substantial confounding from the control variables. For this reason, we reported the results from Model 1 as the main results. All models showed a significant declining time trend in suicide rates (approximately 1–2% per year).

**Table 3:** Conditional Poisson regression (within-region effects).

Variable	Model 1		Model 2		Model 3		Model 4	
	RR (95% CI)	p value	RR (95% CI)	p value	RR (95% CI)	p value	RR (95% CI)	p value
Year	0.986 (0.977–0.995)	0.003	0.992 (0.984–1.000)	0.047	0.991 (0.984–0.999)	0.026	0.986 (0.975–0.996)	0.009
Psychiatric inpatient beds (within)	0.924 (0.881–0.969)	0.001					0.920 (0.847–0.999)	0.048
Outpatient budget percentage (within)			1.002 (0.999–1.006)	0.180			0.999 (0.992–1.006)	0.764
Total psychiatric budget (within)					1.045 (0.963–1.135)	0.294	1.029 (0.943–1.122)	0.525

Rate ratios (95% confidence intervals, CI) from conditional Poisson regression models with county fixed effects examining within-county associations over time. Models control for all time-invariant county characteristics by using each county as its own control. Effects represent how changes in service capacity within counties relate to changes in suicide rates. Clustered standard errors account for within-county correlation. Overdispersion tests showed acceptable fit (dispersion 1.27–1.29, p > 0.30). Psychiatric inpatient beds per 10-unit increase per 100,000 inhabitants; outpatient budget percentage per 1% increase; total psychiatric budget per 1000 SEK increase per capita.

The Bayesian mixed-effects models using MCMC methods confirmed the pattern observed in the fixed-effects analysis (Table 4). Psychiatric beds showed significant protective effects primarily through within-county variation (RR = 0.923, 95% credible interval: 0.866–0.980 in Model 1; RR = 0.919, 95% credible interval: 0.852–0.994 in Model 4), while between-county effects were not statistically significant (95% credible intervals included the null value of 1.0). Neither the total psychiatric budget nor the outpatient budget percentage demonstrated significant associations in either the between or within components, with all credible intervals encompassing 1.0.

**Table 4:** Bayesian MCMC mixed-model Poisson regression (within-between decomposition).

Variable	Model 1	Model 2	Model 3	Model 4
	95% CI for RR	95% CI for RR	95% CI for RR	95% CI for RR
Year	0.986 (0.979, 0.993)	0.992 (0.986, 0.997)	0.991 (0.986, 0.997)	0.985 (0.978, 0.993)
Psychiatric inpatient beds (between)	0.910 (0.802, 1.036)			0.881 (0.756, 1.032)
Psychiatric inpatient beds (within)	0.923 (0.866, 0.980)			0.919 (0.852, 0.994)
Outpatient budget percentage (between)		0.997 (0.986, 1.007)		0.997 (0.985, 1.009)
Outpatient budget percentage (within)		1.002 (0.999, 1.006)		0.999 (0.994, 1.003)
Total psychiatric budget (between)			0.985 (0.789, 1.228)	1.159 (0.868, 1.548)
Total psychiatric budget (within)			1.044 (0.975, 1.116)	1.030 (0.961, 1.107)

Rate ratios (95% credible intervals, CI) from Bayesian mixed-effects Poisson models using Markov Chain Monte Carlo estimation. Between-county effects represent differences in county means; within-county effects represent annual deviations from county means. This decomposition allows simultaneous examination of cross-sectional and longitudinal associations while testing robustness of fixed-effects results. Models run with 4 chains, 2000 iterations (1000 warmup). Psychiatric inpatient beds per 10-unit increase per 100,000 inhabitants; outpatient budget percentage per 1% increase; total psychiatric budget per 1000 SEK increase per capita.

Across all model specifications, suicide rates showed a consistent declining temporal trend, with annual reductions of 1–2% (RR approximately 0.985–0.992). This pattern was robust across different analytical approaches and model specifications.

In the sensitivity analyses, the magnitudes of the estimated within-county rate ratios of psychiatric beds for certain suicides were 0.942–0.969, and for deaths of undetermined intent 0.826–0.877. This is comparable to the rate ratios estimated in the main analyses, though most confidence intervals slightly overlapped the null effect level (Tables S1–S6 in the Supplementary Material). In the pre-pandemic sensitivity analysis restricted to 2015–2019, the within-county effect of bed capacity remained consistent in direction and was somewhat larger in magnitude (RR = 0.88, 95% CI: 0.76–1.01 in the unadjusted model), though statistical significance was not retained, likely reflecting the reduced power from halving the number of observations. Similarly, when Stockholm County was excluded, the effect estimate remained consistent in direction and magnitude (RR = 0.94, 95% CI: 0.86–1.02 in the unadjusted model), with loss of statistical significance again attributable to the reduction in power from removing the largest county from the sample, accounting for 16.8 percent of the total population included in the study.

Based on the within-county estimates from the fixed-effects models, the potential clinical significance of psychiatric bed availability is substantial. Under the assumption of the association being causal, an increase from the 2024 level of 24.2 beds per 100,000 to the 2015 level of 30.2 would be associated with approximately a 5.2% reduction in suicide rates. For Sweden's population of 10.6 million (in 2024) with an average suicide rate of 15.0 per 100,000, this corresponds to approximately 83 fewer suicides annually.

## Discussion

This nationwide ecological study found that higher psychiatric inpatient bed availability was associated with lower suicide mortality across Swedish counties between 2015 and 2024. In the adjusted within-county models, an increase of 10 beds per 100,000 inhabitants was associated with approximately 7.6% reduction in suicide rates. The robustness of the inverse association between psychiatric bed availability and suicide rates across methodologically distinct approaches hints at a potential protective effect of psychiatric inpatient care, and the absence of significant associations for budget variables across model specifications suggests that bed capacity may be a particularly important structural factor associated with suicide outcomes. Suicide remains a major and persistent public health concern in Sweden and globally.^1^, ^2^, ^3^ Our findings suggest that system-level factors, such as the progressive decrease of psychiatric inpatient capacity, may partially explain why national suicide rates have declined only modestly in recent decades. This study contributes novel evidence from a universal healthcare setting, extending prior research.

The reduction in psychiatric inpatient bed capacity observed during the study period occurred within the context of broader structural reforms in the Swedish health system rather than a single policy decision. Over the past decade, national policy has increasingly emphasised strengthening primary care and expanding outpatient and community-based mental health services, as formalised in the “Close Care” reform aimed at improving accessibility and continuity of mental health care^25^ In parallel, legislative changes such as the Act on Coordination of Discharge from Inpatient Care (SFS 2017:612) have aimed to facilitate earlier discharge and improve coordination between hospital and community services.^25^ National clinical guidelines consistently recommend outpatient management as first-line treatment for most psychiatric conditions, reserving inpatient care primarily for acute deterioration, severe symptoms.^26^

These developments reflect a broader structural transformation in the organisation of mental health care rather than clearly reflecting changes in clinical need. Over recent decades, mental health systems in Sweden and other high-income countries have progressively shifted away from institutional models toward outpatient and community-based care, consistent with international policy frameworks and national system reforms.^27^^,^^28^ At the same time, Swedish supervisory authorities have reported that shortages of available inpatient beds are frequently driven by workforce constraints and difficulties maintaining adequate staffing levels, which limit the number of beds that can be kept operational in practice.^29^ Together, these developments represent a sustained reorganisation of psychiatric care, in which inpatient treatment remains essential for acute crises but plays a more targeted role within a predominantly outpatient-oriented system.

The question of whether, and to what extent, psychiatric inpatient care can decrease suicide rates is somewhat contentious, and there is no established consensus view. Admissions to psychiatric inpatient care are commonly used when patients are perceived as having an acute increased risk of suicide because it can offer a measure of immediate protection. Inpatient care is also used to ascertain adequate treatment of severe affective or psychotic episodes, which, if untreated, may increase the risk of suicide. It is by now a well-established fact that suicide rates are particularly high immediately following discharge.^30^^,^^31^ However, the mechanisms underlying this elevated risk remain incompletely understood and likely reflect a complex interaction of illness severity, crisis resolution, and continuity of care rather than the duration of inpatient treatment alone. A recent meta-analysis by Tai et al.^32^ confirmed substantial post-discharge suicide risk but did not identify duration-related factors, such as length of follow-up, as primary explanatory variables. These findings suggest that post-discharge suicide risk is likely influenced by a complex interaction of clinical severity, illness trajectory, and continuity of care rather than any single structural factor. In this context, inpatient bed capacity may influence how inpatient care is used within the health system, including admission thresholds and timing of discharge. While inpatient care can provide safety, stabilization, and intensive support during acute suicidal crises, it may also involve challenging transitions between inpatient and outpatient care. Recent individual-level studies have demonstrated heterogeneous associations between hospitalization and subsequent suicide risk, suggesting that the effects of inpatient care vary across patient subgroups and clinical circumstances.^33^^,^^34^ These findings highlight that inpatient bed capacity represents a structural characteristic of mental health systems whose population-level associations with suicide mortality may reflect multiple interacting clinical and system-level mechanisms. As the rates of psychiatric hospital beds have continued to decline in most high-income countries even after the deinstitutionalization period, concerns are raised that the reduced inpatient capacity may delay or prevent admission for those with the highest need. Patients may instead be managed in less intensive settings or discharged prematurely, which may contribute to increased vulnerability during transitions of care.

The lack of consensus on the potential preventive effects of psychiatric hospital beds on suicide is in part due to the conflicting evidence from the published scientific literature. Early research on the consequences of the international deinstitutionalization from the 1960s to the 1990s was mostly conducted in the Nordic countries and tended to focus on schizophrenia. A Danish and a Swedish study, from 1993 to 2000, respectively, both found evidence for an increase in suicide mortality following deinstitutionalization (Ösby, Mortensen), while two Finnish studies from 2002 to 2007, respectively, failed to find such an association in earlier data periods, and found an inverse association in several diagnostic groups in later data (Salokangas och Pirkola 2007). While these studies were methodologically crude by today's standards, research using more sophisticated methods has continued to provide conflicting evidence. In a 2018 ecological study on data from Hong Kong, Lee and colleagues found no overall association between psychiatric hospital beds and suicide rates in 1999–2015.^35^

The studies described above assess overall suicide rates based on psychiatric hospital bed rates, measured either implicitly or explicitly. Furthermore, the Nordic studies all focused on individual-level data, which cannot capture effects on individuals not included in the study. The employment of within-region or within-state modelling on population-level data constitutes a much stronger research design, increasing statistical power and controls for unmeasured regional characteristics that may confound the analyses. The most influential research comes from the United States, where two key studies employ such research designs, though they reach conflicting conclusions. Using data from 1982 to 1998, Yoon and Bruckner^11^ reported that within-state reductions in public psychiatric beds were associated with increased suicide mortality, particularly in settings with limited community mental-health resources, implying that downsizing may elevate risk. In contrast, Gibbons et al.,^12^ analysing data from 1999 to 2013, found that between-state association, rather than within-state association, explained the overall association between psychiatric hospital beds and suicide rates. These results suggest that there are other factors not taken into account–such as community resources, service organization, and policy differences–that might be associated with both psychiatric hospital beds and suicide rates on a state-level. This highlights the methodological vulnerability even of this more sophisticated research design. Moreover, the U.S. context—with its mixed public–private bed structure and lack of universal coverage—differs substantially from the publicly dominated mental-health systems in most high-income countries, limiting generalisability. A novel simulation-based approach presented by Atkinson et al.^13^ suggests that suicide mortality increases when psychiatric bed availability falls below approximately 25 beds per 100,000 inhabitants, but is unaffected by higher bed rates than that. However, as a simulation study, these results rely on multiple assumptions about patient behavior and care pathways that may not reflect real-world complexity. While European mental-health policy has appropriately emphasised the expansion of community psychiatry,^6^ our findings indicate that insufficient attention to maintaining inpatient capacity may have unintended consequences for suicide risk.

Recent U.S. studies have examined the individual-level effects of psychiatric hospitalization among patients presenting with suicidal behaviours using advanced causal inference methods. Goldman-Mellor et al.,^33^ analysing administrative claims data for patients treated after deliberate self-harm, found no clear overall evidence that hospitalization reduced subsequent suicide risk after adjustment for measured confounding, although treatment effects varied across subgroups. Similarly, Ross et al.,^34^ using precision treatment modelling in a large clinical dataset of patients with suicidal behaviours, reported heterogeneous estimated effects of hospitalization, with potential benefit in some high-risk groups and no benefit or possible harm in others. These studies address the effectiveness of hospitalization as a clinical intervention at the individual level. In contrast, the present study evaluates associations between regional inpatient bed capacity and suicide mortality at the population level, reflecting structural characteristics of health-care systems rather than treatment effects for specific patients.

The current state of research on the issue at hand is fragmented. Most studies do not attempt to control for confounding, some studies are limited to data for selected patient groups, and there are no rigorous studies that provide adequate control for potential confounding. Furthermore, two of the more sophisticated studies are based on data from the U.S., which differs substantially from most high-income countries in its emphasis on private care services and lack of universal coverage.^36^^,^^37^ The present study helps clarify these discrepancies by employing robust methods for inference in ecological research. First, it applies a fixed-effects design that addresses unmeasured confounding at the county level, as well as including control variables reflecting total psychiatric budget spending as well as psychiatric outpatient capacity. Second, it leverages within-country regional variation in a publicly dominated health-care system with universal coverage, a context that closely reflects the organisation of mental-health services across most high-income countries. Third, it employs nationwide, high-quality register data with complete population coverage. Taken together, these methodological and contextual strengths provide strong and broadly generalisable evidence of an inverse association between psychiatric inpatient capacity and suicide mortality.

Several limitations should be considered when interpreting the present results. Although population data were obtained from Statistics Sweden's continuous administrative register and are considered highly accurate, a small degree of uncertainty due to registration delays or inaccuracies cannot be entirely excluded. While the robust statistical methods control for much of the potential confounding, there remains a possibility for residual confounding, which is unavoidable even in quasi-experimental observational research. Second, psychiatric bed availability is a structural indicator. It does not capture variation in care quality, staffing stability, ward safety practices, or follow-up care intensity, all of which can influence suicide risk. The sensitivity analyses yielded comparable results, though several of those analyses were not statistically significant. We interpret this as an issue of statistical power since all of the analyses pointed in the same direction and yielded statistically significant results in the main analysis. However, it is not clear whether there is a differential effect of psychiatric bed availability on certain and uncertain suicides. The analysis was based on aggregate inpatient bed capacity and did not distinguish between beds used for voluntary and involuntary psychiatric care. In Sweden, both types of care are delivered within the same inpatient units without formal bed allocation by legal status. It is possible that changes in bed capacity may differentially affect voluntary and involuntary admissions, which could not be examined in this ecological study. Future research should explore whether the observed association is stronger for specific patient groups or service configurations and examine interactions between inpatient capacity, crisis pathways, and community-based care.

Despite these limitations, the findings have important implications for policy and service planning, especially in high-income countries with universal coverage. Although Sweden has a tax-funded universal health-care system, similar reductions in psychiatric inpatient capacity have occurred in many high-income countries, suggesting that the observed associations may be relevant to comparable health system contexts. Ensuring sufficient inpatient psychiatric capacity should be considered a central component of national and regional suicide-prevention strategies. Monitoring bed availability and real-time access indicators may offer early warnings of elevated suicide risk associated with capacity constraints. Future work should examine potential threshold effects, identify patient groups who benefit most from increased capacity, and assess how inpatient availability interacts with continuity of care after discharge.

The findings suggest that maintaining sufficient psychiatric inpatient capacity should be considered when planning national and regional suicide-prevention efforts. Ensuring timely admission for individuals in acute suicidal crises may help reduce system strain, improve safety during transitions of care, and prevent avoidable deaths. Monitoring of bed availability and real-time access indicators may provide an early warning of increased suicide risk arising from capacity constraints.

Our findings indicate that greater psychiatric inpatient bed availability is inversely associated with suicide mortality at the population level. Using a nationwide dataset and a rigorous statistical modeling approach rarely applied in this field, the study provides evidence that accessible inpatient care may represent an important structural element of effective suicide-prevention systems. Because patterns of bed supply, service organization, and population risk profiles are broadly similar across high-income countries, these results have wide international relevance. Safeguarding and strengthening inpatient capacity may therefore be important for achieving national suicide-prevention goals in many comparable health-care systems.

## Contributors

All authors conceived the study. JB acquired the data and conducted the statistical analyses. SL drafted the introduction, abstract, and discussion sections; JB drafted the methods and results. All authors contributed to the development of the research questions, interpretation of the findings, and critical revision of the manuscript. All authors approved the final version of the manuscript and agree to be accountable for all aspects of the work.

## Data sharing statement

The aggregated data used in this study are publicly available from national Swedish authorities (Statistics Sweden, the National Board of Health and Welfare, and the Swedish Association of Local Authorities and Regions). No individual-level data were accessed. The statistical code used for the analyses is available from the corresponding author upon reasonable request.

Link to the publicly available data:

https://sdb.socialstyrelsen.se/if_dor/val.aspx

https://skr.se/halsaochsjukvard/ekonomiochavgiftersjukvard/ekonomiochverksamhetsstatistiksjukvard.7872.html.

## Declaration of interests

The authors declare no conflicts of interest.
