# Supplementary material for: Psychiatric inpatient bed capacity and suicide mortality in Sweden: a nationwide ecological study
Source: Lancet Reg Health Eur. 2026 Apr 8;66:101675. doi: 10.1016/j.lanepe.2026.101675 (PMC13186040; doi:10.1016/j.lanepe.2026.101675)
Supplement: Supplementary Material [file mmc1.docx]

# Supplementary material

## Analyses with certain suicides (X60-X84)

| **Table S1**. Pooled cross-sectional Poisson Regression (Between-Regions Effects). Certain suicides (X60-X64). | | | | |
| --- | --- | --- | --- | --- |
|  | *Unadjusted* | | *Adjusted* | |
| Variable | RR (95% CI) | p value | RR (95% CI) | p value |
| Psychiatric inpatient beds | 0.845 (0.769-0.929) | 0.003 | 0.896 (0.786-1.022) | 0.122 |
| Outpatient budget percentage | 0.987 (0.980-0.994) | 0.002 | 0.989 (0.977-1.001) | 0.102 |
| Total psychiatric budget | 0.797 (0.675-0.941) | 0.016 | 1.091 (0.828-1.437) | 0.546 |

| **Table S2**. Conditional Poisson Regression (Within-Region Effects). Certain suicides (X60-X64). | | | | | | | | |
| --- | --- | --- | --- | --- | --- | --- | --- | --- |
|  | *Model 1* | | *Model 2* | | *Model 3* | | *Model 4* | |
| Variable | RR (95% CI) | p value | RR (95% CI) | p value | RR (95% CI) | p value | RR (95% CI) | p value |
| Year | 1.000 (0.991-1.008) | 0.931 | 1.002 (0.993-1.011) | 0.674 | 1.002 (0.993-1.01) | 0.704 | 0.997 (0.986-1.009) | 0.626 |
| Psychiatric inpatient beds (within) | 0.969 (0.893-1.052) | 0.457 |  |  |  |  | 0.942 (0.851-1.042) | 0.242 |
| Outpatient budget percentage (within) |  |  | 0.999 (0.995-1.004) | 0.714 |  |  | 0.997 (0.991-1.003) | 0.260 |
| Total psychiatric budget (within) |  |  |  |  | 1.022 (0.945-1.105) | 0.584 | 1.027 (0.936-1.126) | 0.575 |

| **Table S3**. Bayesian MCMC Mixed-Model Poisson Regression (Within-Between Decomposition). Certain suicides (X60-X64). | | | | |
| --- | --- | --- | --- | --- |
|  | *Model 1* | *Model 2* | *Model 3* | *Model 4* |
| Variable | 95% CI for RR | 95% CI for RR | 95% CI for RR | 95% CI for RR |
| Year | 1.000 (0.992, 1.008) | 1.002 (0.996, 1.008) | 1.002 (0.995, 1.008) | 0.997 (0.988, 1.006) |
| Psychiatric inpatient beds (between) | 0.903 (0.774, 1.054) |  |  | 0.890 (0.748, 1.059) |
| Psychiatric inpatient beds (within) | 0.969 (0.903, 1.038) |  |  | 0.943 (0.861, 1.032) |
| Outpatient budget percentage (between) |  | 0.993 (0.981, 1.004) |  | 0.991 (0.979, 1.004) |
| Outpatient budget percentage (within) |  | 0.999 (0.995, 1.003) |  | 0.997 (0.991, 1.002) |
| Total psychiatric budget (between) |  |  | 0.966 (0.748, 1.268) | 1.198 (0.881, 1.673) |
| Total psychiatric budget (within) |  |  | 1.021 (0.945, 1.101) | 1.027 (0.949, 1.109) |

## Analyses with death of undetermined intent (Y10-Y34)

| **Table S4**. Pooled cross-sectional Poisson Regression (Between-Regions Effects). Undetermined intent (Y10-Y34). | | | | |
| --- | --- | --- | --- | --- |
|  | *Unadjusted* | | *Adjusted* | |
| Variable | RR (95% CI) | p value | RR (95% CI) | p value |
| Psychiatric inpatient beds | 1.106 (0.888-1.376) | 0.380 | 0.83 (0.631-1.09) | 0.199 |
| Outpatient budget percentage | 1.019 (1.006-1.033) | 0.011 | 1.026 (0.999-1.053) | 0.077 |
| Total psychiatric budget | 1.398 (1.036-1.888) | 0.042 | 1.082 (0.592-1.977) | 0.800 |

| **Table S5**. Conditional Poisson Regression (Within-Region Effects). Undetermined intent (Y10-Y34). | | | | | | | | |
| --- | --- | --- | --- | --- | --- | --- | --- | --- |
|  | *Model 1* | | *Model 2* | | *Model 3* | | *Model 4* | |
| Variable | RR (95% CI) | p value | RR (95% CI) | p value | RR (95% CI) | p value | RR (95% CI) | p value |
| Year | 0.941 (0.922-0.961) | <.001 | 0.954 (0.934-0.975) | <.001 | 0.952 (0.932-0.973) | <.001 | 0.946 (0.921-0.972) | <.001 |
| Psychiatric inpatient beds (within) | 0.826 (0.687-0.993) | 0.042 |  |  |  |  | 0.877 (0.656-1.172) | 0.375 |
| Outpatient budget percentage (within) |  |  | 1.01 (0.997-1.023) | 0.134 |  |  | 1.006 (0.987-1.024) | 0.542 |
| Total psychiatric budget (within) |  |  |  |  | 1.06 (0.955-1.175) | .275 | 0.98 (0.789-1.217) | 0.855 |

| **Table S6**. Bayesian MCMC Mixed-Model Poisson Regression (Within-Between Decomposition). Undetermined intent (Y10-Y34). | | | | |
| --- | --- | --- | --- | --- |
|  | *Model 1* | *Model 2* | *Model 3* | *Model 4* |
| Variable | 95% CI for RR | 95% CI for RR | 95% CI for RR | 95% CI for RR |
| Year | 0.941 (0.927, 0.956) | 0.954 (0.942, 0.967) | 0.952 (0.94, 0.964) | 0.945 (0.929, 0.962) |
| Psychiatric inpatient beds (between) | 0.892 (0.665, 1.202) |  |  | 0.827 (0.559, 1.209) |
| Psychiatric inpatient beds (within) | 0.826 (0.715, 0.95) |  |  | 0.874 (0.728, 1.041) |
| Outpatient budget percentage (between) |  | 1.011 (0.988, 1.035) |  | 1.019 (0.989, 1.049) |
| Outpatient budget percentage (within) |  | 1.01 (1.002, 1.018) |  | 1.005 (0.995, 1.016) |
| Total psychiatric budget (between) |  |  | 1.011 (0.602, 1.712) | 0.971 (0.466, 2.04) |
| Total psychiatric budget (within) |  |  | 1.064 (0.921, 1.237) | 0.985 (0.853, 1.143) |

## Influence of Stockholm County

Stockholm county accounted for approximately 16.8% of the total study population, making it by far the largest county in the sample. To assess the potential influence of Stockholm county on the overall results, the primary fixed-effects models were re-estimated excluding Stockholm county (n=188 observations, 19 counties).

In the unadjusted model, the within-county effect of psychiatric bed capacity was RR=0.94 (95% CI: 0.86–1.02, p=0.15) when Stockholm was excluded, compared to RR=0.92 (95% CI: 0.88–0.97, p<0.001) in the full sample. In the fully adjusted model, the corresponding estimates were RR=0.95 (95% CI: 0.88–1.02, p=0.17) and RR=0.92 (95% CI: 0.85–1.00, p=0.048) respectively.

The direction and magnitude of the effect estimate remained consistent across both specifications when Stockholm was excluded, though statistical significance was not retained, likely reflecting the substantial reduction in statistical power when removing the largest county from a sample of only 20 units. These findings are further corroborated by the county-level forest plot (Figure S1), which shows that Stockholm's individual posterior estimate is unremarkable in magnitude relative to other counties. Taken together, these results suggest that the overall finding is not driven by Stockholm county, but that Stockholm contributes meaningfully to the precision of the pooled estimate.

## **Pre-COVID Period (2015–2019)**

To assess whether the results were influenced by the COVID-19 pandemic or related changes in psychiatric service utilisation and suicide reporting during 2020–2024, the primary fixed-effects models were re-estimated restricting the sample to the pre-pandemic period (2015–2019, n=100 observations, 20 counties).

In the unadjusted model, the within-county effect of psychiatric bed capacity was RR=0.88 (95% CI: 0.76–1.01, p=0.07) in the pre-pandemic period, compared to RR=0.92 (95% CI: 0.88–0.97, p<0.001) in the full sample. In the fully adjusted model, the corresponding estimates were RR=0.89 (95% CI: 0.71–1.11, p=0.31) and RR=0.92 (95% CI: 0.85–1.00, p=0.048) respectively.

The effect estimates remained consistent in direction and were somewhat larger in magnitude in the pre-pandemic period, though statistical significance was not retained, which is expected given that restricting to five years halves the number of observations and substantially reduces statistical power. These findings suggest that the main results are not driven by pandemic-related changes in suicide rates or psychiatric service utilisation.

## Technical Appendix

This appendix provides technical details on model specifications and supplementary analyses conducted to ensure transparency and methodological rigour. Formal model specifications with notation are provided for all three analytical approaches used in the main paper. Supplementary analyses address the robustness of the Bayesian models through standard convergence diagnostics, assess potential sources of bias through examination of temporal autocorrelation and collinearity, and explore heterogeneity in the primary effect estimate across counties.

## Specification of Regression Models

#### Pooled Cross-Sectional Poisson Regression

The formula for the fully adjusted pooled cross-sectional Poisson regression presented in Table 1 in the paper is as follows:

$$log(E[{suicides}_{i}])=log({population}_{i})+\beta_{0}+\beta_{1}hospital {beds}_{i}+\beta_{2}{outpatient budget}_{i}+\beta_{3}{total budget}_{i}$$

Where $i$ indexes county. The outcome variable suicides denotes the combined count of certain and uncertain suicides for county $i$ over 2015–2024, with ${population}_{i}$ (sum of population of county $i$ over the same period) included as an offset to model rates. Hospital beds denotes the mean number of available psychiatric inpatient beds per 100,000 inhabitants (though divided by 10 in order to provide more interpretable estimates), outpatient budget denotes the mean proportion of the total psychiatric budget allocated to outpatient services, and total budget denotes the mean total psychiatric budget per capita, all averaged over 2015–2024 for county i. A quasi-Poisson specification was used to account for overdispersion, introducing a dispersion parameter $\varphi$ such that:

$Var({suicides}_{i}) = \varphi\times E[{suicides}_{i}]$

with standard errors scaled accordingly. The unadjusted models were identical in structure but each included only one exposure variable. Note that calendar year was not included in the pooled cross-sectional models, as time is collapsed across the study period in this specification.

#### Conditional Poisson Regression

The full conditional Poisson regression model presented in Table 2 in the paper was specified as follows:

$$log(E[{suicides}_{ij}])=log({population}_{ij})+\alpha_{i}+\beta_{1}{year}_{j}+\beta_{2}hospital {beds}_{ij}+\beta_{3}{outpatient budget}_{ij}+\beta_{4}{total budget}_{ij}$$

Where $i$ indexes county and $j$ indexes year. The outcome variable ${suicides}_{ij}$ denotes the combined count of certain and uncertain suicides for county $i$ in year $j$, with ${population}_{ij}$ included as an offset to model rates. The county-specific fixed effect $\alpha_{i}$ absorbs all time-invariant county-level characteristics, observed and unobserved, such that each county serves as its own control. Year is included as a continuous variable to account for secular trends in suicide rates. Hospital beds denotes the available psychiatric inpatient beds per 100,000 inhabitants (divided by 10 in order to provide more interpretable estimates), outpatient budget denotes the proportion of the total psychiatric budget allocated to outpatient services, and total budget denotes the total psychiatric budget per capita, all measured annually for county $i$ in year $j$.

County fixed effects were estimated via conditional maximum likelihood, and clustered standard errors at the county level were used to account for within-county correlation over time. The unadjusted models were identical in structure but each included only one exposure variable alongside the fixed effect and year trend.

#### Bayesian MCMC Mixed-Model Poisson Regression

The Bayesian Mixed-Effects Model (Within-Between Decomposition) presented in Table 3 was specified as follows:

$$log(E[{suicides}_{ij}])=log({population}_{ij})+\beta_{0}+\beta_{1}{year}_{j}+\beta_{2}hospital {beds between}_{i}+\beta_{3}hospital {beds within}_{ij}+\beta_{4}{outpatient budget between}_{i}+\beta_{5}{outpatient budget within}_{ij}+\beta_{6}{total budget between}_{i}+\beta_{7}{total budget within}_{ij}+\mu_{i}$$

Where $i$ indexes county and $j$ indexes year. The outcome variable ${suicides}_{ij}$ denotes the combined count of certain and uncertain suicides for county i in year j, with ${population}_{ij}$ included as an offset to model rates. Year is included as a continuous variable to account for secular trends in suicide rates. Each exposure variable is decomposed into two components: a between-county component (${hospital beds between}_{i}$, ${outpatient budget between}_{i}$, and ${total budget between}_{i}$), representing the county mean over the study period and capturing stable cross-sectional differences between counties, and a within-county component (${hospital beds within}_{ij}$, ${outpatient budget within}_{ij}$, and ${total budget within}_{ij}$), representing the annual deviation from that county mean and capturing longitudinal change within counties over time. Hospital beds is expressed per 100,000 inhabitants (divided by 10 to provide more interpretable estimates), outpatient budget denotes the proportion of the total psychiatric budget allocated to outpatient services, and total budget denotes the total psychiatric budget per capita. The county-level random effect $\mu_{i}\sim N(0, \sigma^{2})$ accounts for residual between-county variation. Models were estimated using Markov Chain Monte Carlo methods via the *brms* package in R, with 4 chains of 2000 iterations each (1000 warmup). The unadjusted models were identical in structure but each included only one exposure variable alongside the year trend and random effect.

## Supplementary Analyses and Diagnostics

#### Autocorrelation and Collinearity

In order to assess temporal autocorrelation in model residuals, lag-1 autocorrelation was computed within each county separately using Pearson residuals from the fully adjusted fixed-effects model, and summarized across counties. The mean within-county lag-1 autocorrelation was -0.127 (SD = 0.288, range: -0.577 to 0.574), indicating no systematic temporal autocorrelation in the residuals on average. While some individual counties showed moderate positive or negative autocorrelation, these did not follow a consistent directional pattern, suggesting that residual autocorrelation is unlikely to have systematically biased the standard errors or coefficient estimates. It should be noted that individual county autocorrelation estimates are inherently noisy given that each county contributes only nine time points, which likely accounts for much of the observed variation across counties.

To assess collinearity between predictor variables in the fully adjusted models, variance inflation factors (VIFs) were computed using an equivalent Poisson regression model. All VIF values were low, ranging from 1.24 (year) to 1.53 (total psychiatric budget), well below the conventional threshold of 5, indicating no meaningful collinearity between psychiatric bed capacity, outpatient budget percentage, and total psychiatric budget. Collinearity is therefore unlikely to have biased the coefficient estimates or inflated their standard errors.

#### Bayesian Model Diagnostics

To demonstrate the robustness of the Bayesian mixed-effects models, we report standard MCMC convergence diagnostics for all four models in Table S1. For each parameter we report the potential scale reduction factor (R-hat), bulk and tail effective sample size (ESS), and lag-1 autocorrelation of posterior draws averaged across chains.

| **Table S1**. Bayesian MCMC Mixed-Model Regression Diagnostics | | | | |  |  |
| --- | --- | --- | --- | --- | --- | --- |
| Parameter | | Rhat | Bulk ESS | Tail ESS | Mean Lag1 ACF | Max Lag1 ACF |
| Model 1 | |  |  |  |  |  |
|  | Intercept | 1.005 | 847 | 1484 | 0.519 | 0.555 |
|  | Year | 1.000 | 4029 | 3343 | -0.049 | 0.011 |
|  | Psychiatric inpatient beds (between) | 1.006 | 851 | 1391 | 0.521 | 0.553 |
|  | Psychiatric inpatient beds (within) | 1.000 | 3315 | 2788 | 0.049 | 0.133 |
|  | County random intercept | 1.005 | 1055 | 1315 | 0.481 | 0.525 |
| Model 2 | |  |  |  |  |  |
|  | Intercept | 1.003 | 1300 | 1612 | 0.467 | 0.528 |
|  | Year | 1.000 | 7396 | 2943 | -0.302 | -0.244 |
|  | Outpatient budget percentage (between) | 1.003 | 1328 | 1610 | 0.460 | 0.515 |
|  | Outpatient budget percentage (within) | 1.001 | 5452 | 2856 | -0.165 | -0.122 |
|  | County random intercept | 1.004 | 815 | 1511 | 0.494 | 0.559 |
| Model 3 | |  |  |  |  |  |
|  | Intercept | 1.002 | 1201 | 1736 | 0.497 | 0.554 |
|  | Year | 1.001 | 5131 | 2797 | -0.194 | -0.163 |
|  | Total psychiatric budget (between) | 1.002 | 1223 | 1835 | 0.489 | 0.551 |
|  | Total psychiatric budget (within) | 1.001 | 3323 | 2405 | -0.009 | 0.059 |
|  | County random intercept | 1.006 | 1040 | 1254 | 0.502 | 0.542 |
| Model 4 | |  |  |  |  |  |
|  | Intercept | 1.004 | 1885 | 2375 | 0.321 | 0.399 |
|  | Year | 1.000 | 3133 | 2719 | 0.087 | 0.139 |
|  | Psychiatric inpatient beds (between) | 1.003 | 1235 | 1591 | 0.457 | 0.521 |
|  | Psychiatric inpatient beds (within) | 1.000 | 2622 | 2726 | 0.162 | 0.246 |
|  | Outpatient budget percentage (between) | 1.003 | 1552 | 1902 | 0.368 | 0.421 |
|  | Outpatient budget percentage (within) | 1.000 | 3266 | 2909 | 0.005 | 0.018 |
|  | Total psychiatric budget (between) | 1.003 | 1483 | 1979 | 0.449 | 0.477 |
|  | Total psychiatric budget (within) | 1.001 | 3518 | 2831 | 0.010 | 0.130 |
|  | County random intercept | 1.004 | 1057 | 1875 | 0.422 | 0.466 |

All R-hat values were at or below 1.006, indicating satisfactory convergence across all models and parameters. Bulk ESS was above 400 for all parameters, though some between-county parameters and intercepts showed values slightly below 1000 (range: 815–1885), reflecting the limited number of counties (n=20) available to inform these estimates. Tail ESS was above 1000 for all parameters across all models, supporting the reliability of the reported credible intervals. Within-county parameters of primary inferential interest showed consistently high ESS values. Lag-1 autocorrelation was low for all within-county parameters (range: -0.165 to 0.246), indicating efficient sampling for the parameters of primary interest, while between-county parameters and intercepts showed moderate autocorrelation (range: 0.321 to 0.559), consistent with their somewhat lower Bulk ESS values.

#### Random Slope Models

To examine whether the association between psychiatric inpatient bed capacity and suicide mortality varied across counties, we fitted random slope models allowing the within-county effect of bed capacity to vary across counties. Both the unadjusted (Model 1) and fully adjusted (Model 4) specifications were estimated using Bayesian Poisson mixed-effects models with the same prior and sampling settings as the main analyses.

Both models converged satisfactorily, with all R-hat values ≤1.003 and adequate ESS for all parameters. A single divergent transition was observed in each model, which is unlikely to materially affect the results given the overall stability of the chains.

The standard deviation of the random slope for within-county bed capacity was 0.067 (95% CI: 0.002–0.215) in the unadjusted model and 0.087 (95% CI: 0.004–0.254) in the fully adjusted model. The lower bounds of both credible intervals barely exceeded zero, suggesting limited evidence for meaningful heterogeneity in the beds effect across counties. The small number of counties (n=20) precludes precise estimation of between-county variation in slopes, and the correlation between random intercepts and slopes was poorly identified in both models, with credible intervals spanning nearly the entire possible range.

The within-county coefficient for bed capacity remained consistent in direction and magnitude with the main analyses, at −0.080 (95% CI: −0.164 to 0.003) in the unadjusted model and −0.096 (95% CI: −0.210 to 0.003) in the fully adjusted model, though credible intervals marginally included zero, likely reflecting the reduced precision inherent in random slope specifications with a limited number of higher-level units.

#### Bayesian County-Level Estimates and Heterogeneity

County-specific posterior estimates of the within-county beds effect were extracted from the fully adjusted Bayesian random slope model to assess heterogeneity in the association between psychiatric inpatient bed capacity and suicide mortality across counties. These estimates represent the model-adjusted association between within-county changes in bed capacity and suicide mortality for each county, net of secular time trends, budget variables, and between-county differences.

County-level estimates are presented in Figure S1. Nineteen of 20 counties showed a negative point estimate, indicating a broadly consistent protective direction of the beds effect across the country. The sole exception was Gävleborg, which showed a point estimate marginally above zero (0.008) with a wide credible interval (−0.170 to 0.331), consistent with a null or weakly protective effect rather than a harmful one. Point estimates among the remaining counties ranged from −0.046 (Dalarna) to −0.214 (Östergötland), reflecting moderate heterogeneity in the magnitude of the effect. Three counties had 95% credible intervals excluding zero: Östergötland, Uppsala, and Västerbotten. The remaining counties showed wide credible intervals consistent with limited statistical power at the individual county level. The overall pooled estimate from the fully adjusted model (−0.096, 95% CI: −0.210 to 0.003) is shown for reference.


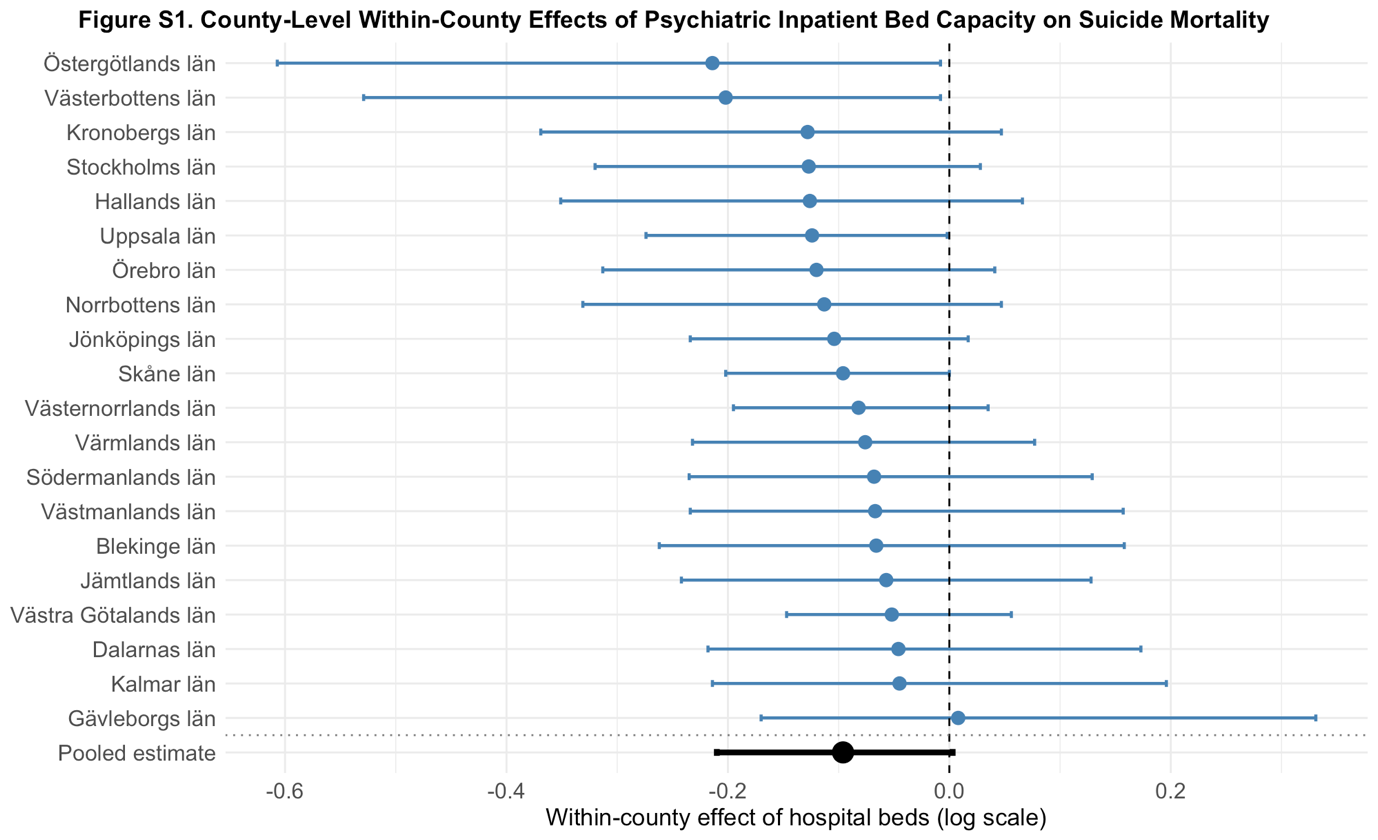


The consistency of direction across counties provides additional support for the robustness of the main findings, suggesting that the protective association between bed capacity and suicide mortality is not driven by any single county or region.

## Sensitivity analyses

#### Influence of Stockholm County

Stockholm county accounted for approximately 16.8% of the total study population, making it by far the largest county in the sample. To assess the potential influence of Stockholm county on the overall results, the primary fixed-effects models were re-estimated excluding Stockholm county (n=188 observations, 19 counties).

In the unadjusted model, the within-county effect of psychiatric bed capacity was RR=0.94 (95% CI: 0.86–1.02, p=0.15) when Stockholm was excluded, compared to RR=0.92 (95% CI: 0.88–0.97, p<0.001) in the full sample. In the fully adjusted model, the corresponding estimates were RR=0.95 (95% CI: 0.88–1.02, p=0.17) and RR=0.92 (95% CI: 0.85–1.00, p=0.048) respectively.

The direction and magnitude of the effect estimate remained consistent across both specifications when Stockholm was excluded, though statistical significance was not retained, likely reflecting the substantial reduction in statistical power when removing the largest county from a sample of only 20 units. These findings are further corroborated by the county-level forest plot (Figure S1), which shows that Stockholm's individual posterior estimate is unremarkable in magnitude relative to other counties. Taken together, these results suggest that the overall finding is not driven by Stockholm county, but that Stockholm contributes meaningfully to the precision of the pooled estimate.

#### **Sensitivity Analysis: Pre-COVID Period (2015–2019)**

To assess whether the results were influenced by the COVID-19 pandemic or related changes in psychiatric service utilisation and suicide reporting during 2020–2024, the primary fixed-effects models were re-estimated restricting the sample to the pre-pandemic period (2015–2019, n=100 observations, 20 counties).

In the unadjusted model, the within-county effect of psychiatric bed capacity was RR=0.88 (95% CI: 0.76–1.01, p=0.07) in the pre-pandemic period, compared to RR=0.92 (95% CI: 0.88–0.97, p<0.001) in the full sample. In the fully adjusted model, the corresponding estimates were RR=0.89 (95% CI: 0.71–1.11, p=0.31) and RR=0.92 (95% CI: 0.85–1.00, p=0.048) respectively.

The effect estimates remained consistent in direction and were somewhat larger in magnitude in the pre-pandemic period, though statistical significance was not retained, which is expected given that restricting to five years halves the number of observations and substantially reduces statistical power. These findings suggest that the main results are not driven by pandemic-related changes in suicide rates or psychiatric service utilisation.
